# Multiscale neural dynamics in sleep transition volatility across age scales: a multimodal EEG-EMG-EOG analysis of temazepam effects

**DOI:** 10.1007/s11357-024-01342-6

**Published:** 2024-09-14

**Authors:** Parikshat Sirpal, William A. Sikora, Hazem H. Refai

**Affiliations:** 1 School of Electrical and Computer Engineering, University of Oklahoma, Gallogly College of Engineering, Norman, OK 73019 USA; 2 School of Biomedical Engineering, University of Oklahoma, Gallogly College of Engineering, Norman, OK 73019 USA

**Keywords:** Aging, Sleep physiology, Sleep transition, GARCH modeling, Volatility, Multimodal neuroimaging, Tensor-based analysis, Time–frequency decomposition

## Abstract

Recent advances in computational modeling techniques have facilitated a more nuanced understanding of sleep neural dynamics across the lifespan. In this study, we tensorize multiscale multimodal electroencephalogram (EEG), electromyogram (EMG), and electrooculogram (EOG) signals and apply Generalized Autoregressive Conditional Heteroskedasticity (GARCH) modeling to quantify interactions between age scales and the use of pharmacological sleep aids on sleep stage transitions. Our cohort consists of 22 subjects in a crossover design study, where each subject received both a sleep aid and a placebo in different sessions. To understand these effects across the lifespan, three evenly distributed age groups were formed: 18–29, 30–49, and 50–66 years. The methodological framework implemented here employs tensor-based machine learning techniques to compute continuous wavelet transform time–frequency features and utilizes a GARCH model to quantify sleep signal volatility across age scales. Support Vector Machines are used for feature ranking, and our analysis captures interactions between signal entropy, age, and sleep aid status across frequency bands, sleep transitions, and sleep stages. GARCH model results reveal statistically significant volatility clustering in EEG, EMG, and EOG signals, particularly during transitions between REM and non-REM sleep. Notably, volatility was higher in the 50–66 age group compared to the 18–29 age group, with marked fluctuations during transitions from deep sleep to REM sleep (standard deviation of 0.35 in the older group vs. 0.30 in the 18–29 age group, *p* < 0.05). Statistical comparisons of volatility across frequency bands, age scales, and sleep stages highlight significant differences attributable to sleep aid use. Mean conditional volatility parameterization of the GARCH model reveals directional influences, with a causality index of 0.75 from frontal to occipital regions during REM sleep transition periods. Our methodological framework identifies distinct neural behavior patterns across age groups associated with each sleep stage and transition, offering insights into the development of targeted interventions for sleep regularity across the lifespan.

**Supplementary Information:**

The online version contains supplementary material available at 10.1007/s11357-024-01342-6.

## Introduction

Neural mechanisms and brain dynamics governing sleep transitions provide insight into sleep physiology across the lifespan. Multiple transition states within distinct neurophysiological sleep stages including rapid eye movement (REM) and non-REM (NREM) sleep [1–6]. These stages are linked to age-related changes in cognitive function, memory consolidation, and overall brain health [7, 8]. Stage transition dynamics vary, are age-dependent, and are influenced by pharmacological interventions [9, 10]. The EEG signal can be represented as a manifold in state space, where pseudo-stationary dynamics of the EEG neural manifold represent distinct trajectories corresponding to varying sleep states [11, 12]. Delta waves (0.5–4 Hz) are most prominent during deep sleep (slow-wave sleep, stages 3 and 4 of NREM sleep) and are relevant to restorative sleep; theta waves (4–8 Hz) are seen during light sleep (stages 1 and 2 of NREM sleep) and REM sleep, associated with the transition from wakefulness to sleep and with dreaming [13]. Alpha waves (8–12 Hz) appear when a person is awake but relaxed and can also indicate disrupted or shallow sleep during the transition into sleep [3–5, 12]. Beta waves (12–30 Hz), associated with active thinking and alertness, can appear during REM sleep or indicate arousal and wakefulness [4, 6]. Gamma waves (30–100 Hz), involved in higher cognitive functions, are less commonly analyzed in sleep studies but can appear during REM sleep linked to complex processing and dreaming [4]. Sleep spindles (11–16 Hz), brief bursts of activity during stage 2 of NREM sleep, play a role in memory consolidation and protecting sleep by inhibiting sensory processing and slow wave activity (SWA) (0.5–2 Hz) represents the deepest stage of sleep, important for restorative processes and known to decrease with age [14–16]. By modeling these dynamics, we attempt to gain insight into the neural mechanisms underlying sleep transitions and their volatility across different age groups. The primary aim of this study is to evaluate the effects of temazepam, a pharmacological sleep aid, on aging-associated changes in sleep neural behavior patterns. By examining how temazepam modulates sleep transitions and neural dynamics across different age groups, this study seeks to provide insights that could inform targeted interventions for improving sleep health in aging populations.

Aging is associated with significant sleep pattern changes including reductions in sleep duration, changes in sleep stage distribution, and increased nighttime awakenings [17, 18]. These alterations are not merely signs of aging but are linked with various age-related neurological disorders, such as dementia and Parkinson’s disease [17–19]. Recent advances in neuroimaging and signal processing have enabled more precise investigations of these dynamics, particularly, multimodal approaches involving electroencephalography (EEG), electromyography (EMG), and electrooculography (EOG). This multimodal approach has provided new insights into the intricate patterns of sleep neural activity associated with sleep [20, 21] and enabled detailed characterization of electrical activity in the brain, muscle movements, and eye movements, respectively. Parameterizing EEG-EMG-EOG multimodal signal volatility to examine sleep transitions can be used as input to develop targeted interventions to potentially mitigate negative impacts of aging on sleep. In this work, we leverage data derived from the Expanded Sleep-EDF database in which sleep telemetry multimodal EEG, EMG, and EOG signals were collected from 22 subjects (males (*n* = 7) and females (*n* = 15)) [22, 23]. To facilitate multiscale neural dynamics analysis, we first organized the multimodal EEG-EMG-EOG signals in a tensor format [24, 25]. Compared to traditional methods, tensor-based modeling offers several advantages, as it enables the simultaneous analysis of multiple dimensions (i.e., time, frequency, and channels), thus preserving modality interdependencies [24, 26] [24–26, 26, 27]. This is superior to methods that analyze each modality in isolation, which may overlook critical cross-modal interactions. Moreover, tensor decomposition techniques can efficiently reduce dimensionality while retaining essential features, facilitating more accurate and interpretable models.

Following tensor formation, we applied wavelet-based time–frequency analysis to the multimodal EEG-EMG-EOG sleep tensor, helping to demarcate features, and the quantification of signal volatility. Specifically, wavelet analysis for time–frequency decomposition helps to observe neural activities across multiple frequency bands [28, 29]. Following time–frequency signal decomposition, we perform feature selection and form a feature vector that will be used as input into a Generalized Autoregressive Conditional Heteroskedasticity (GARCH) model to compute the volatility in tensor dynamics [30–33]. By exploring the volatility in neural dynamics during sleep and its modulation by age and pharmacological interventions, our results have implications across age scales, suggesting potential pathways through which aging-related changes in sleep might be mitigated or managed. This offers parameters for developing effective interventions that enhance the sleep quality and life quality across age scales.

In this study, we analyzed retrospective data that utilized temazepam as a pharmacological intervention. Temazepam, a benzodiazepine commonly prescribed for short-term management of insomnia, was selected for the original dataset due to its established efficacy and safety profile in clinical practice. While the focus of this research is to investigate the neural dynamics and volatility associated with sleep transitions under the influence of temazepam, the findings are intended to provide a foundation for future studies that may examine the effects of other hypnotic drugs. This analysis emphasizes the importance of understanding how pharmacological interventions modulate sleep, particularly in the context of aging-associated changes. We propose a data-driven workflow for the first time that incorporates multivariate features of sleep dynamics and quantitatively analyzes sleep stage transitions and neural signal volatility. This approach aims to provide a comprehensive analysis of sleep transitions and develop targeted interventions to mitigate the negative impacts of aging on sleep.

The main contributions of our work are as follows:

1. Development of a data-driven workflow: Integrating multiscale, multimodal EEG, EMG, and EOG data to characterize sleep transitions and assess the effects of aging and sleep aids on sleep stage dynamics.
2. Advanced analysis techniques: Employing wavelet analysis for time–frequency decomposition and Generalized Autoregressive Conditional Heteroskedasticity (GARCH) models to explore and quantify the volatility in neural dynamics during sleep transitions.
3. Quantitative assessment: Identifying significant differences in neural dynamics and volatility between younger and elderly adults across various sleep stages, with a focus on transitions from deep sleep to REM sleep with and without the use of sleep pharmacotherapy.
4. Predictive relationships: Applying GARCH models to determine the directional influences and relationships between different brain regions during sleep transitions.

The “Methods” section introduces the experimental setup and EEG data processing techniques used in our work and describes tensor construction, continuous wavelet analysis, and the GARCH modeling approach employed in this work. The “Results” section discusses the experimental results and the “Discussion” and “Conclusion” sections include the discussion and conclusion. The supplemental section provides further analyses of mean conditional variance and MANOVA interactions. 

## Methods

### Data

Sleep telemetry multimodal EEG, EMG, and EOG signals collected from 22 Caucasian males (*n* = 7) and females (*n* = 15) [22, 23] were used in this study and participants were divided into three evenly distributed 18–29 $(n= 8)$, 30–49 $(n= 7)$, and 50–66 year$(n=7)$ old groups. Demographic details are presented in Table 1, with subjects ranked by age. Multimodal data was collected to examine the impact of temazepam effects on subjects who are otherwise healthy and not taking other medications but encounter difficulty falling asleep and includes full night duration (i.e., 9 h) polysomnographic recordings in a controlled hospital setting over two nights. A sleep aid, temazepam, was administered during the first night, followed by a placebo on the subsequent night. Signals represented in 30-s epochs were scored by experts according to the Rechtschaffen and Kales (R&K) scale, classifying sleep into different stages based on EEG, EOG, and EMG activity [34]. A power analysis was conducted using a significance level of 0.05 and a power of 0.80, across age groups as well as the full cohort. Effect size estimates, specifically focusing on mean differences in sleep stage transitions between the temazepam and placebo subject populations, were examined, ensuring that the sample size of 22 subjects provided adequate statistical power to detect significant differences with an effect size of 0.5 or greater, thereby validating the robustness of our study’s findings across all age groups.

**Table 1:** Demographic profiles of study subjects including age and sex. The drug night assignment indicates whether the participant received the sleep aid during the first (1) or second session (2)

ID	Age	Sex	Drug night
4	18	Female	2
10	20	Female	2
14	20	Male	2
11	21	Female	1
12	21	Male	2
13	22	Male	1
20	24	Male	2
19	28	Female	1
5	32	Female	1
21	34	Female	1
2	35	Female	1
6	35	Female	2
9	47	Male	1
17	48	Female	2
24	48	Female	2
7	51	Female	2
18	53	Female	1
22	56	Male	2
1	60	Male	2
8	66	Female	1
15	66	Female	2
16	79	Female	1

### Multimodal data processing

All multimodal data were sampled at 100 Hz, with event markers recorded at 1 Hz. Each 30-s epoch was expertly scored according to the R&K scale [22, 23]. Additionally, a physical marker dimension (i.e., “ID,” “M,” “E”) was utilized. Specifically, the “M” marker produced a 2-s deflection from the baseline value. A positive deflection identifies the active telemetry unit (ID = 1 or 2), while a negative deflection indicates an error (“E”) in the telemetry link. Detailed descriptions of the subjects and recordings are available further in [22, 23]. These recordings featured horizontal EOG and EEG signals from “$Fpz-Cz$” and “$Pz-Oz$” channels. The raw EEG, EOG, and EMG data were aligned with the expertly R&K scaled scored data and were partitioned into 30-s epochs. Multimodal data was processed using custom-made scripts in the Python programming language (Python ver. 3.9.1). A neutral virtual reference was applied to EEG recordings via the Reference Electrode Standardization Technique [35, 36]. To estimate brain activity and confirm appropriate SNR, Welch’s power spectral density (PSD) [37] was computed for each EEG channel using a heuristically determined time window duration of 11 s and a corresponding frequency resolution of 0.125 Hz. Logarithmic coordinate plots (log-power vs. log-frequency) were used to estimate brain activity. Utilizing the mean of the power spectral densities obtained for all channels, the global power spectral density was subsequently calculated. Following this, to compute physiologically relevant frequency bands, the data was bandpass filtered between 0.5 and 45 Hz effectively reducing signal noise and artifacts [38, 39]. Frequency band powers were computed in the following physiological EEG frequency bands: slow wave activity, spindle, sigma, delta, theta, alpha, beta, and gamma. EEG frequency envelopes were extracted within the following ranges (delta, 0.5–4 Hz; theta, 4–8 Hz; alpha, 8–13 Hz; beta, 14–30 Hz; gamma, 30–45 Hz; slow wave activity, 0.5–2 Hz; spindle, 12–16 Hz; and sigma, 12–15 Hz) [40]. Utilizing the Parks-McClellan algorithm, optimal Chebyshev finite impulse response filters (FIR) were designed with customized order to minimize error in the pass and stop bands, effectively removing signal drift and 60 Hz noise [41, 42].

Physiological noise, including heartbeat and respiration, was removed from the EEG signal using a high-pass filter with a cutoff frequency of 1.0 Hz. Ocular artifacts within the EEG signals such as eye movements and blinks were removed using fast Independent Component Analysis (ICA) [43, 44]. Specifically, spatial ICA components were extracted from the bandpass filtered EEG data (1–45 Hz), the unmixing ICA matrix was applied, and the components were rendered for visual inspection. Components were evaluated based on their topography and time course and components deemed to be artifacts were identified and removed, and the remaining EEG data was re-referenced. EMG data is band-pass filtered to isolate the frequency range relevant to muscle activity, typically between 20 and 50 Hz [39, 45], removing low-frequency noise, movement artifacts, and baseline drift, as well as high-frequency noise. Following band-pass filtering, the EMG data was rectified and then smoothed using a low-pass filter, with a cutoff frequency of 7.5 Hz, to create a linear envelope that represents the overall muscle activity over time [39, 45]. The root mean square (RMS) of the EMG signal was computed with a window length of 100 ms determined heuristically, to capture the time-varying nature of muscle activation. Additionally, power spectral density (PSD) analysis using the Welch method was computed specifically by segmenting the EMG signal into overlapping windows, applying a window function (i.e., Hamming window), and computing the Fourier transform of each segment [39, 45, 46]. Raw EOG data was first band-pass filtered to isolate the frequency range pertinent to eye movements, between 0.1 and 15 Hz, removing high-frequency noise and low-frequency drift, following which the EOG signal is rectified and smoothed using a low-pass filter, with a cutoff frequency of 5 Hz [47, 48]. To separate EOG components from overlapping EEG data, fast ICA was applied ensuring accurate artifact removal and clean EOG signal extraction [44]. Table 2 below shows the frequencies of interest and their corresponding physiological features in sleep.

**Table 2:** EEG, EMG, and EOG frequency bands of interest in sleep and their significance in sleep

Physiological correlate of sleep stage	Frequency range (Hz)	Description	Significance in sleep
EEG—Delta waves	1–4	Deep sleep	Promotes restorative functions
EEG—Theta waves	4–8	Drowsiness	Memory consolidation may play a role in dreaming
EEG—Alpha waves	8–13	Relaxed wakefulness	Mental processing
EEG—Beta waves	13–30	Alertness	Focused attention
EEG—Gamma waves	30–100	Information processing	Sensory integration
EEG—Slow wave activity (SWA)	0.5–4 (within delta)	Deep sleep	Promotes sleep stability and memory consolidation
EEG—Sleep spindles	11–16 (typically sigma)	NREM Sleep	Memory consolidation, thalamocortical communication
EMG	N/A	Sleep stages	Reduced activity during sleep compared to wakefulness
EOG	N/A	REM sleep	Rapid eye movements

### Tensor formation

Post-processing to facilitate multiscale neural dynamics analysis, the resultant multimodal signals are organized into a tensor (i.e., multidimensional array). Specifically, $X$ denotes the tensor containing our processed data as the following:

1 $$X\in {\mathbb{R}}^{T\times N\times C}$$

where $T$ represents the number of time points, $N$ denotes the modality quantity, and $C$ represents the number of channels for each signal type. Thus, each element ${X}_{T, N, C}$ in the tensor corresponds to the value of signal $n$ at time point $t$ in channel $c$.

Specifically, the data was organized into a 5-dimensional tensor. The first dimension (size, 22) corresponds to the number of subjects included in the study. The second dimension (size, 3) represents the different modalities: electroencephalography (EEG), electrooculography (EOG), and electromyography (EMG). The third dimension (size, 7) captures the number of frequency bands extracted from the EEG-EMG-EOG data using wavelet analysis. This allows for the examination of neural activity within specific frequency ranges relevant to sleep stage transitions (i.e., delta waves for deep sleep, alpha waves for wakefulness). The fourth dimension (size, 100) represents the number of frequency bins within each EEG band. The final dimension (size, T) represents the total number of time points (i.e., epochs) in the recording for each subject. This is calculated by multiplying the recording duration (i.e., 8 h) by the sampling rate (i.e., 100 Hz). For an 8-h recording with a 100 Hz sampling rate, $T = 8\text{ hours }\times 3600\text{ seconds}/\text{hour }\times 100\text{ samples}/\text{second }=\text{ 2,880,000 timepoints}$. This tensor structure facilitates the exploration of multiscale neural dynamics across different modalities and time points, enabling a comprehensive investigation of sleep stage transitions.

### Wavelet transformation, feature selection, and statistical analysis for sleep stage classification

Now, to analyze the tensor data, we apply time–frequency analysis particularly, the continuous wavelet transform (CWT). Due to its optimal time–frequency localization properties, we utilize the Morlet wavelet (Eq. 2) as the mother wavelet in our analysis [29, 49, 50]. Mathematically, the Morlet wavelet is defined as follows:

2 $$\varphi \left(t\right)={\pi }^{-\frac{1}{4}}{e}^{i{\omega }_{0}t}{e}^{-\frac{{t}^{2}}{2}}$$

where ${\omega }_{0}$ is the central frequency of the wavelet. Note that the CWT of a signal, $X\left(t\right)$, using the Morlet wavelet is given by the following:

3 $${W}_{x}\left(a,b\right)=\frac{1}{\sqrt{a}}{\int }_{-\infty }^{\infty }x\left(t\right){\varphi }^{*}\frac{t-b}{a}dt$$

where $a$ and $b$ are the scale and translation parameters, respectively, and ${\varphi }^{*}$ denotes the complex conjugate of the Morlet wavelet. For tensor $X$, the CWT is applied along the time dimension, $T$ producing a time–frequency representation, $W\in {\mathbb{C}}^{A\times B\times N\times C}$, where $A$ and $B$ represent the scale and translation dimensions introduced by the CWT, respectively. Each element ${W}_{a,b,n,c}$ in the tensor $W$ represents the wavelet coefficient for signal $n$ at scale $a$, translation, $b$, and channel $c$. This transformation results in time frequency features that capture the dynamic neural activities across the entire length of the sleep stages.

Next, we apply Generalized Autoregressive Conditional Heteroskedasticity (GARCH) modeling to the time–frequency features post-CWT processing. The standard GARCH (1,1) model and its mathematical formulation are defined in the “GARCH modeling” section. In this context, GARCH models are particularly suitable to capture neural dynamic volatility. To determine sleep transitions, we extracted features from the last 30 s of each sleep stage as this time scale is the minimum length of sleep stages marked in the data. The GARCH model outputs volatility measures that provide insights into the stability or instability of the neural dynamics at different stages of sleep. These measures are further analyzed using mean conditional variance. Following this, multiway ANOVA with Bonferroni correction is performed to determine the statistical significance of GARCH outputs with respect to multimodal variables (i.e., age, sex, drug status, sleep stage, frequency band, sensor location, and modality type) to control for the increased risk of Type I errors when performing multiple statistical tests. In summary, comprehensive CWT-based feature selection identifies distinguishing characteristics between sleep stages and frequency bands (i.e., wake, REM, and non-REM sleep stages (Stages 1–4, SWA, and Spindles). The following sections provide details regarding wavelet transformation and feature selection.

#### Wavelet transformation

The fast Fourier transform provides frequency domain information but fails to preserve time-localized features, in the context of sleep analysis where transitions and short-duration events are significant. Wavelet transformations, in contrast, offer a multi-resolution analysis capability as they decompose signals into frequency components while retaining the time domain, making them particularly suited for identifying transient features and patterns associated with different sleep stages. By applying the continuous wavelet transform (CWT), statistical measures were extracted across different frequency bands and time intervals, enhancing our ability to differentiate between sleep stages. The CWT of a given signal $x\left(t\right)$ is defined as the following:

4 $$W\left(a, b\right)= {\int }_{\left\{-\infty \right\}}^{\left\{\infty \right\}}x\left(t\right){\psi }^{*}\left.\left( \frac{t-b}{a}\right.\right)dt$$

where $\uppsi \left(t\right)$ is the mother wavelet, $a$ is the scale parameter, and $b$ is the translation parameter. These wavelet coefficients capture both the temporal and spectral characteristics of the signal, serving as the initial set of features. Next, we organized these wavelet coefficients into a feature matrix $X$, where each row corresponds to a different time window (i.e., 30 s), and each column corresponds to a different wavelet coefficient. Corresponding sleep stage labels are stored in a separate vector, $y$.

#### Feature selection

The following statistical methods and machine learning techniques were used to select the most discriminative features between different sleep stages. Mutual information was employed to measure the *dependencies* between features and labels. Additionally, we fitted a random forest classifier to compute feature importance based on the Gini impurity or mean decrease in accuracy, where the importance of feature $j$ is given by the following:

5 $${\text{Importance}}\left(j\right)=\frac{1}{{N}_{t}}{\sum }_{t=1}^{{N}_{t}}{I}_{t}\left(j\right)$$

Support Vector Machine (SVM) classifiers with linear kernels were employed, ranking features by the absolute value of their weights $\left|{w}_{j}\right|$ in the decision function. To enhance the feature selection process, Recursive Feature Elimination (RFE) with cross-validation was applied, using SVM as the underlying model. RFE iteratively removes the least important features by fitting the SVM model and ranking features by importance until the desired number of features is reached. This process generated a binary matrix indicating feature selection for distinguishing among sleep stages. Matrix $B$ has rows corresponding to sleep stages and columns to features (time in seconds), where $B\left({s}_{i},f\right)=1$ if feature $f$ is selected for distinguishing the sleep stage pair $\left({s}_{i},{s}_{j}\right)$, and $B\left({s}_{i},f\right)=0$ if not selected. This binary feature selection data was visualized using a heatmap, highlighting the most discriminative features selected for distinguishing specific pairs of sleep stages. In addition to traditional statistical features, we also included wavelet parameters (i.e., entropy, mean, and energy). These parameters were derived from continuous wavelet transform (CWT) and provide critical insights into the time–frequency characteristics of the neural signals. The mathematical formulations for these wavelet features are as follows:

- Wavelet signal energy

It represents the power of the signal within specific frequency bands, scaled by its importance indicator, ${b}_{i}$, and is calculated as follows:

6 $$E={\sum }_{i=1}^{N}{\left|{W}_{i}\right|}^{2}\times {b}_{i}$$

where ${W}_{i}$ is the wavelet coefficient, $N$ is the number of coefficients, and $b$ is the importance.

- Wavelet signal mean

It provides an overall measure of signal magnitude over time, scaled by its importance ${b}_{i}$, and computed as follows:

7 $${\text{Mean}}=\frac{1}{N}{\sum }_{i=1}^{N}{W}_{i}\times {b}_{i}$$

where ${W}_{i}$ is the wavelet coefficient, $N$ is the number of coefficients, and ${b}_{i}$ is the importance indicator.

- Wavelet signal entropy

It quantifies the complexity and unpredictability of the signal, modified to include importance $b$. It is defined as follows:

8 $${\text{Entropy}}=-{\sum }_{i=1}^{N}{p}_{i}\text{log}\left({p}_{i}\times {b}_{i}\right)$$

where ${p}_{i}$ represents the normalized wavelet coefficient, ${p}_{i}=\frac{{\left|{W}_{i}\right|}^{2}}{{\sum }_{i=1}^{N}{\left|{W}_{i}\right|}^{2}}$, and $b$ is the importance.

- Shannon entropy

It measures the unpredictability or randomness of a set of values, modified to include importance $b$. For a discrete random variable $X$ with possible values ${x}_{1},{x}_{2},\dots ,{x}_{n}$ and a probability mass function $P\left(X\right)$, the Shannon entropy is defined as follows:

9 $$H\left(X\right)=-{\sum }_{i=1}^{n}P\left({x}_{i}\right){\mathit{log}}_{a}\left(P\left({x}_{i}\right)\times {b}_{i}\right)$$

where: 

$H\left(X\right)$ is the Shannon entropy of the random variable, $X$; $P\left({x}_{i}\right)$ is the probability of occurrence of the value, ${x}_{i}$; and $a$ is the base of the logarithm, which determines the unit of entropy; and ${b}_{i}$ represents the importance factor. These wavelet parameters, including wavelet signal entropy and Shannon entropy, are integrated into the feature selection process to ensure a comprehensive representation of the neural signals. This approach enhances the ability to distinguish among sleep stages by capturing the dynamic transitions and complexity of the signals and the importance indicator matrix *B* with respect to the wavelet features ensures that the importance of each feature is considered, providing a weighted representation of the data and distinguishing among sleep stages by highlighting the most relevant features.

#### Statistical analysis

After CWT feature extraction, univariate and multivariate statistical testing was performed to determine statistical significance across age scales, frequency bands, and sleep status. Analysis of variance (ANOVA) was employed to identify significant differences in individual features across different age groups, frequency bands, and sleep aid conditions. Post-hoc testing using Tukey’s Honesty Significant Difference was used to determine group differences. Additionally, *t*-tests were performed to compare the means of features between two groups (i.e., sleep aid versus placebo), and chi-square tests were utilized to evaluate the association between categorical variables, i.e., age groups and sleep stages. Complementing the univariate analyses, multivariate statistical tests were conducted to understand the combined effect of multiple features and their interactions. Multivariate analysis of variance (MANOVA) was applied to test for significant differences across groups for multiple dependent variables simultaneously, providing insights into how combinations of features varied across age groups, frequency bands, and sleep aid status.

### GARCH modeling

The CWT extracted features were employed in a Generalized Autoregressive Conditional Heteroskedasticity (GARCH) modeling to capture volatility patterns across the tensor of EEG-EOG-EMG signals and probe into the underlying transition states. This allows for a detailed understanding of the dynamic properties of neural signals during sleep, highlighting the effects of aging and sleep aids on sleep architecture and neural stability. The GARCH model is particularly suitable to highlight aging and sleep aids on sleep architecture as the model accounts for time-varying volatility within neural signals [31, 32, 51, 52]. Unlike other models that assume constant variance over time, GARCH models can adapt to periods of varying volatility, making them ideal for capturing the inherent fluctuations in neural activity. Additionally, GARCH models are advantageous over other methods as they can model volatility clustering, where periods of high volatility tend to be followed by high volatility and periods of low volatility tend to be followed by low volatility [51, 52], which is often observed in physiological data. The standard GARCH (1,1) model was used, defined by the following:

10 $${y}_{t}= \mu + {\epsilon }_{t}$$

11 $${\epsilon }_{t}= {\sigma }_{t}{z}_{t}$$

12 $${\sigma }_{t}^{2}= {\alpha }_{0}+ {\alpha }_{1}{\epsilon }_{\left\{t-1\right\}}^{2}+ {\beta }_{1}{\sigma }_{\left\{t-1\right\}}^{2}$$

Here, ${y}_{t}$ represents the observed time series data at time,$t$, which in this context are the features extracted from the tensor. The term $\mu$ denotes the mean of the series, ${\epsilon }_{t}$ is the error term at time $t$, and ${\sigma }_{t}^{2}$ is the conditional variance (i.e., volatility) of the series at time $t$.

The parameter ${\alpha }_{0}$ is the constant term, and ${\alpha }_{1}$ measures the impact of past squared errors with respect to volatility at time, $t$, and ${\beta }_{1}$ is the persistence of past volatility on current volatility. The term ${z}_{t}$ represents a sequence of independent and identically distributed ($i.i.d.$) standard normal variables. For each selected feature, the GARCH (1,1) model was fitted to the time dimension of the tensor, thereby allowing for an estimate of the volatility associated with each feature. The GARCH model parameters $\left(( {\alpha }_{0}), ({\alpha }_{1}), ({\beta }_{1})\right)$ were estimated using maximum likelihood estimation [30, 31]. Once the model was fit, we extracted the conditional variances ${\sigma }_{t}^{2}$ as measures of volatility for each feature. By applying the GARCH model to the features extracted from the tensor, we quantify the volatility of neural signals across different sleep stages, age groups, and conditions of sleep aid use. This approach allowed us to identify periods of increased volatility, corresponding to changes in neural dynamics. The GARCH model outputs were further analyzed to determine their statistical significance with respect to multimodal variables. This involved performing multiway ANOVA with Bonferroni correction to assess the effects of age, sleep stage, frequency band, sensor location, and modality type on the volatility measures. We further analyzed the mean conditional volatility across different age groups, sleep stages, and conditions (REM/non-REM, sleep aid versus placebo, and specific sleep activities such as sleep spindles and slow wave activity) to probe their effects on neural dynamics.

#### Mean conditional volatility

The mean conditional volatility (MCV), derived from GARCH modeling, measures the expected variability of neural signals given past information. In our work, MCV quantifies fluctuations in neural signal activity across different sleep stages, age groups, and experimental conditions, providing an indirect measure of neural signal stability and predictability, identifying transitions in neural states across sleep stages, age groups, and responses to external interventions (i.e., pharmacological agents, specifically sleep aids). To further elucidate the relationship between age and neural dynamics, we compute the mean volatility and its variation across different age groups and sleep stages for both placebo and drug conditions (Supplemental Figure S1). The standard GARCH (1,1) model was used, defined above. For each combination of age group, sleep stage, and condition (placebo or drug), mean volatility was calculated by averaging the volatility values over the time intervals for each feature within the specified groups:

13 $$\text{Mean volatility}=\frac{1}{n}{\sum }_{i=1}^{n}{\sigma }_{t,i}^{2}$$

where ${\upsigma }_{t,i}^{2}$ is the volatility at time $t$ for the ${i}^{th}$ feature, and $n$ is the number of features.

### Causality analysis of volatility measures

To further analyze the causality in neural dynamics across different age groups, frequency bands, and sleep stages, we computed the causality index using a mathematical formulation that quantifies the directional influence between neural signals. The causality index, $CI$, for a given frequency band,$f$; sleep stage,$s$; and age group, $a$, was calculated as follows:

14 $$C{I}_{f,s,a}=\frac{1}{N}{\sum }_{i=1}^{N}I\left({X}_{i}\to {Y}_{i}\right)$$

where $I\left({X}_{i}\to {Y}_{i}\right)$ represents the information flow from signal ${X}_{i}$ to signal ${Y}_{i}$ within a specific time window, and $N$ is the total number of time windows analyzed. The time windows utilized here are defined based on the duration of the sleep stage $s$ and are chosen to ensure sufficient temporal resolution for capturing the dynamics of the frequency band $f$ within each age group $a$, quantifying the directional influence of one signal onto another over multiple time windows, thereby providing a robust measure of causality that accounts for temporal variations in neural activity across different sleep stages and age groups. To measure the extent of this influence, we compute the transfer entropy [53, 54] between signal pairs. Transfer entropy is defined as follows:

15 $${T}_{X\to Y}={\sum }_{x,y}p\left({y}_{t+1},{y}_{t},{x}_{t}\right)\text{log}\frac{p\left({y}_{t+1}|{y}_{t},{x}_{t}\right)}{p\left({y}_{t+1}|{y}_{t}\right)}$$

where $p\left({y}_{t+1},{y}_{t},{x}_{t}\right)$ is the joint probability distribution of $Y$ at time $( t+1 ), ( Y )$ at time$t$, and $X$ at time$t$. The term $\left(p\left({y}_{t+1}|{y}_{t},{x}_{t}\right)\right)$ is the conditional probability of $( Y )$ at time $( t+1 )$ given $( Y )$ at time $( t )$ and $( X )$ at time$( t )$, and $(p\left({y}_{t+1}|{y}_{t}\right))$ is the conditional probability of $( Y)$ at time $( t+1 )$ given $( Y )$ at time$( t )$. This determines the directional dependency of $( Y )$ on$( X )$, quantifying the influence of $( X )$ on$( Y )$. Transfer entropy informs the directionality of the information transfer, unlike traditional correlation measures which only capture the strength of the relationship without directional information [53].

Causality analysis was performed across frequency bands (delta, 0.5–4 Hz; theta, 4–8 Hz; alpha, 8–12 Hz; beta, 12–30 Hz; gamma, 30–100 Hz), sleep stages (wake, stage 1, stage 2, stage 3, stage 4, REM), and age groups. To perform this analysis, we first extracted features from the EEG-EOG-EMG tensor using continuous wavelet transform (CWT) and subsequently applied the GARCH model to quantify the volatility of these features (“Wavelet transformation” and “Feature selection” sections). The causality index was then computed on the volatility measures obtained from the GARCH model, allowing us to assess the influence of different neural signals on each other within the specified contexts of age, frequency bands, and sleep stages. Figure 4 shows these interactions, highlighting the mean causality index and its variability (shaded regions) across different conditions. Notably, delta and theta bands show stronger and more stable directional influences during deep sleep stages (stage 3 and REM) in older age groups. This stability contrasts with the greater variability observed in alpha and beta bands, suggesting less consistent stability.

**Fig. 1:**
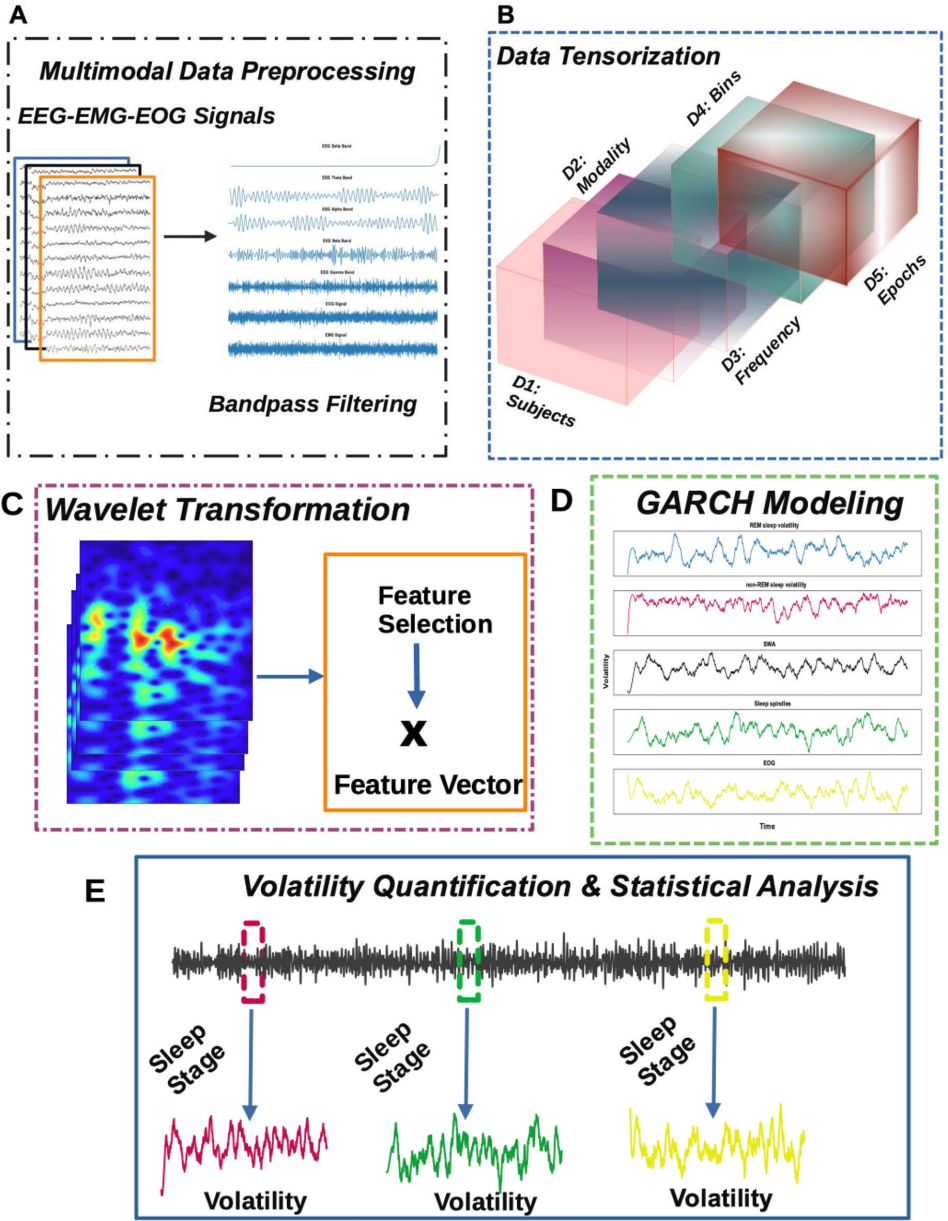
Methodological framework for sleep study analysis. **A** Multimodal data preprocessing including bandpass filtering of EEG, EMG, and EOG signals. **B** Data tensorization taking into consideration dimensions such as subjects (D1), modality (D2), frequency (D3), bins (D4), and epochs (D5). **C** Wavelet transformation and feature selection process. **D** GARCH modeling of different sleep states: REM sleep, non-REM sleep, slow-wave activity (SWA), sleep spindles, and EOG signals. **E** Volatility quantification and statistical analysis highlighting transitions between different sleep states

Figure 1 below shows the framework used to process, bandpass filter, and tensorize; perform time–frequency computations via continuous wavelet transformation; apply the GARCH model; and compute volatility quantification and statistical analysis.

## Results

### Statistical evaluation of EEG frequency variations across age groups and treatment conditions

**Fig. 2:**
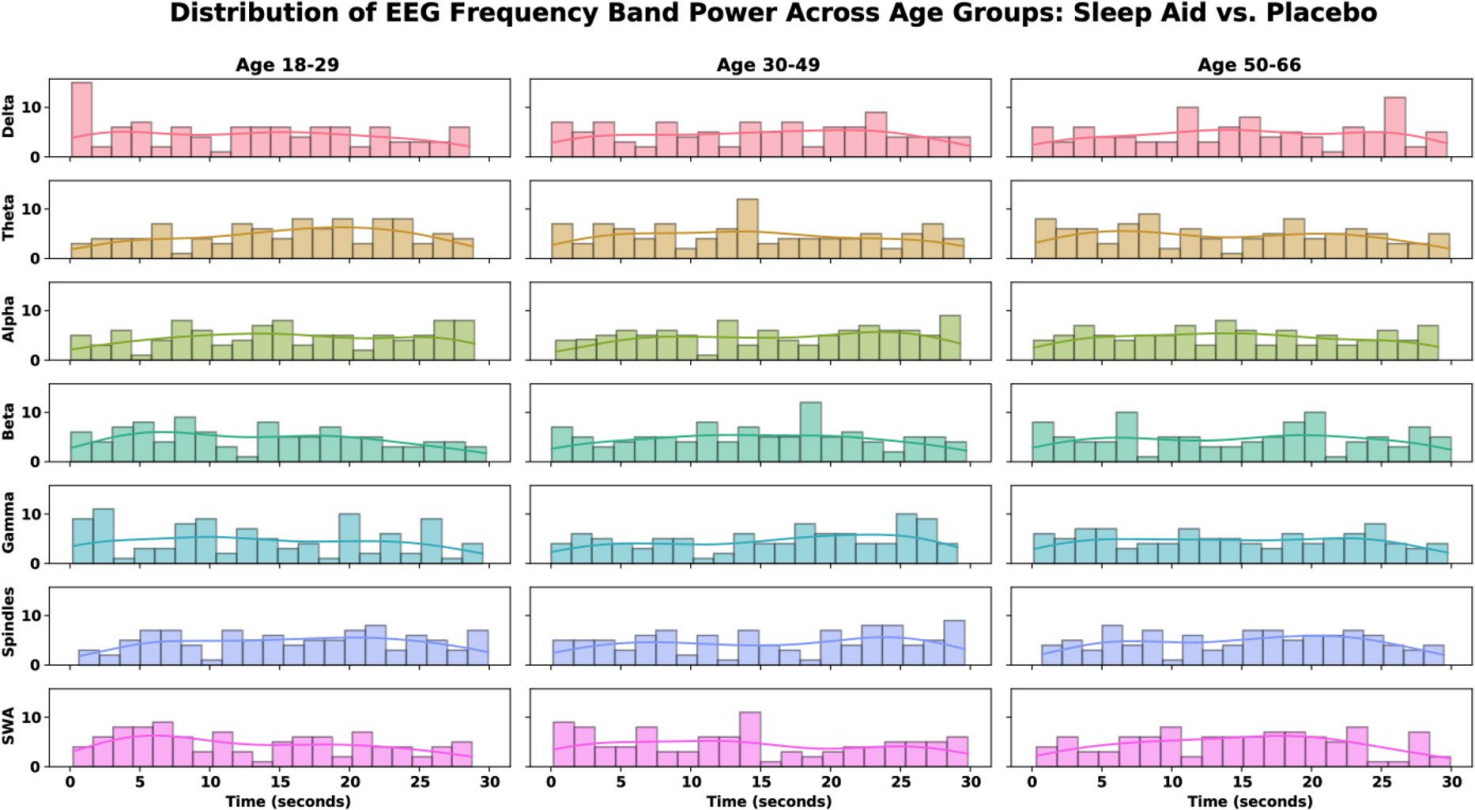
EEG frequency band power distribution across age groups: sleep aid vs. placebo. EEG frequency band power across different age groups comparing the sleep aid group and the placebo group. The frequency bands analyzed include delta, theta, alpha, beta, gamma, spindles, and slow wave activity (SWA). Each subplot shows the distribution of EEG power over time (0–30 s) for a specific frequency band and age group

**Fig. 3:**
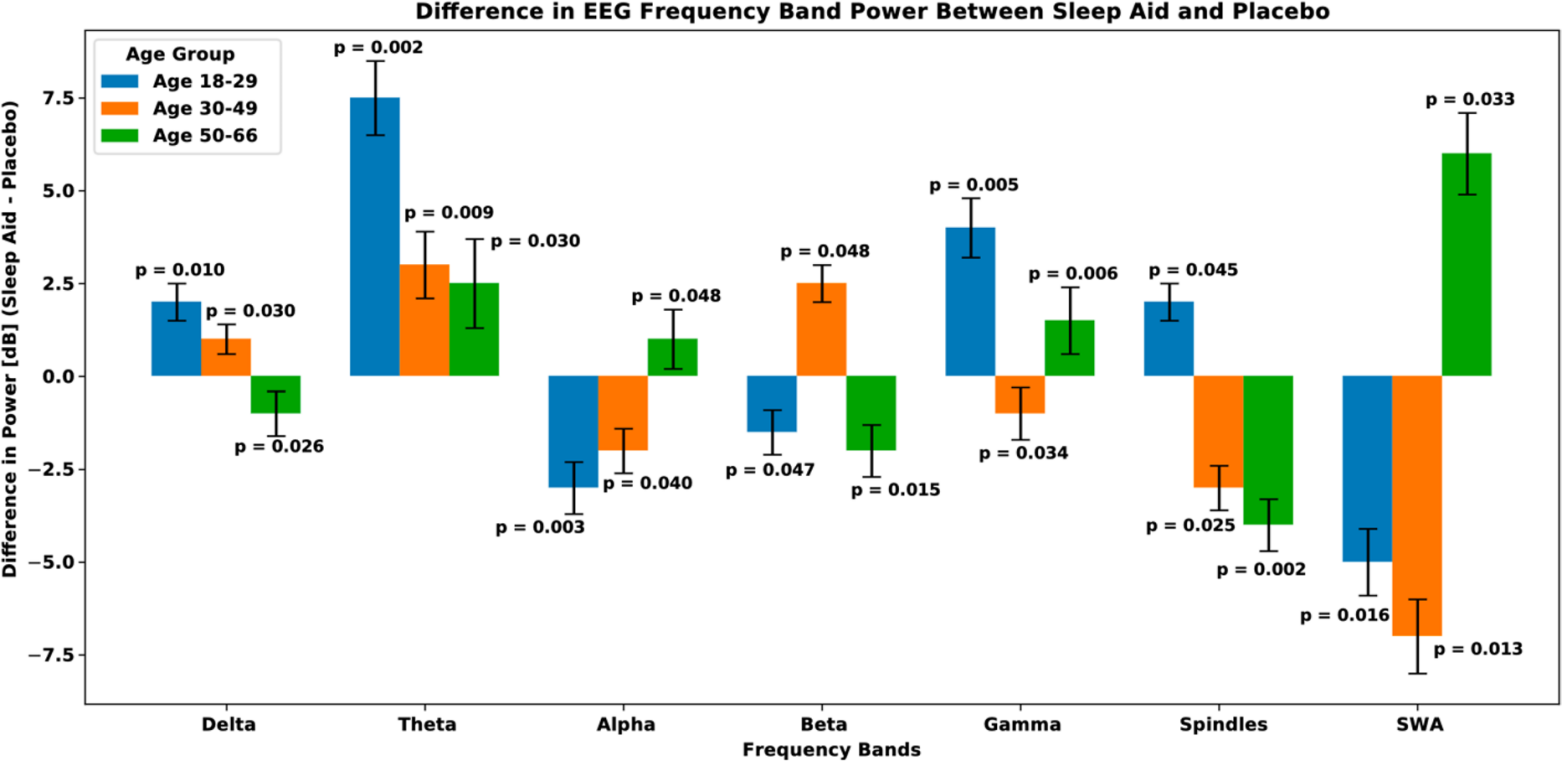
Difference in EEG frequency band power between sleep aid and placebo. The difference in power (dB) between sleep aid and placebo conditions across various EEG frequency bands (delta, theta, alpha, beta, gamma, spindles, and SWA) for three age groups (18–29, 30–49, and 50–66). *p*-values are annotated, and error bars represent the standard deviation of the power differences

Figure 2 below highlights the effects of sleep aid use versus placebo over 30-s intervals with respect to EEG frequency band power across different age groups. Significant age-related declines in delta band power, spindle density, and slow wave activity (SWA) are observed in the placebo group, indicating a reduction in deep sleep quality and duration in older adults. In contrast, the sleep aid group shows preserved delta power, enhanced spindle activity, and maintained SWA across age groups, suggesting that sleep aids may help mitigate age-related declines in sleep quality and stability. To assess the impact of sleep aid intervention on age-related changes in EEG frequency bands, we calculated the distribution of EEG frequency band power across different age groups and treatment conditions, characterizing the influence of sleep medication on neural activity across the lifespan (Fig. 2). We further computed the differences in EEG frequency band power between sleep aid and placebo conditions (Fig. 3), accompanied by statistical testing, to assess the impact of the sleep aid on neural activity, allowing for a detailed comparison of its effects across frequency bands and age groups.

### CWT feature selection and GARCH modeling for multimodal sleep transition state analysis

In this section, we present the results of our continuous wavelet transform (CWT) feature selection and subsequent GARCH modeling methodologies applied in the context of multimodal sleep transition state analysis. Our analysis focused on identifying relevant features from the EEG, EMG, and EOG signals to characterize sleep state transitions accurately. By leveraging CWT, we extracted time–frequency features, which were then subsequently utilized in a GARCH model to capture the dynamic volatility across different sleep stages. Among the time–frequency features extracted, we considered wavelet signal energy, mean, and entropy. Wavelet signal energy represents the power of the signal within specific frequency bands, providing insights into the amplitude variations of neural activity. The mean of the wavelet coefficients gives an overall measure of signal magnitude over time. Wavelet signal entropy quantifies the complexity and unpredictability of the signal [28, 29]. Once extracted, Shannon entropy was computed for all features. The most informative feature across all subjects was wavelet signal entropy as the Shannon entropy values were the highest indicating most complexity. Higher Shannon entropy values for wavelet signal entropy suggest greater variability and richness in the signal’s information content, making it particularly sensitive to changes and transitions in neural states [54, 55]. This sensitivity is essential for capturing the nuanced and transient phenomena that characterize sleep stage transitions, which might be less apparent in features with lower entropy values.

**Fig. 4:**
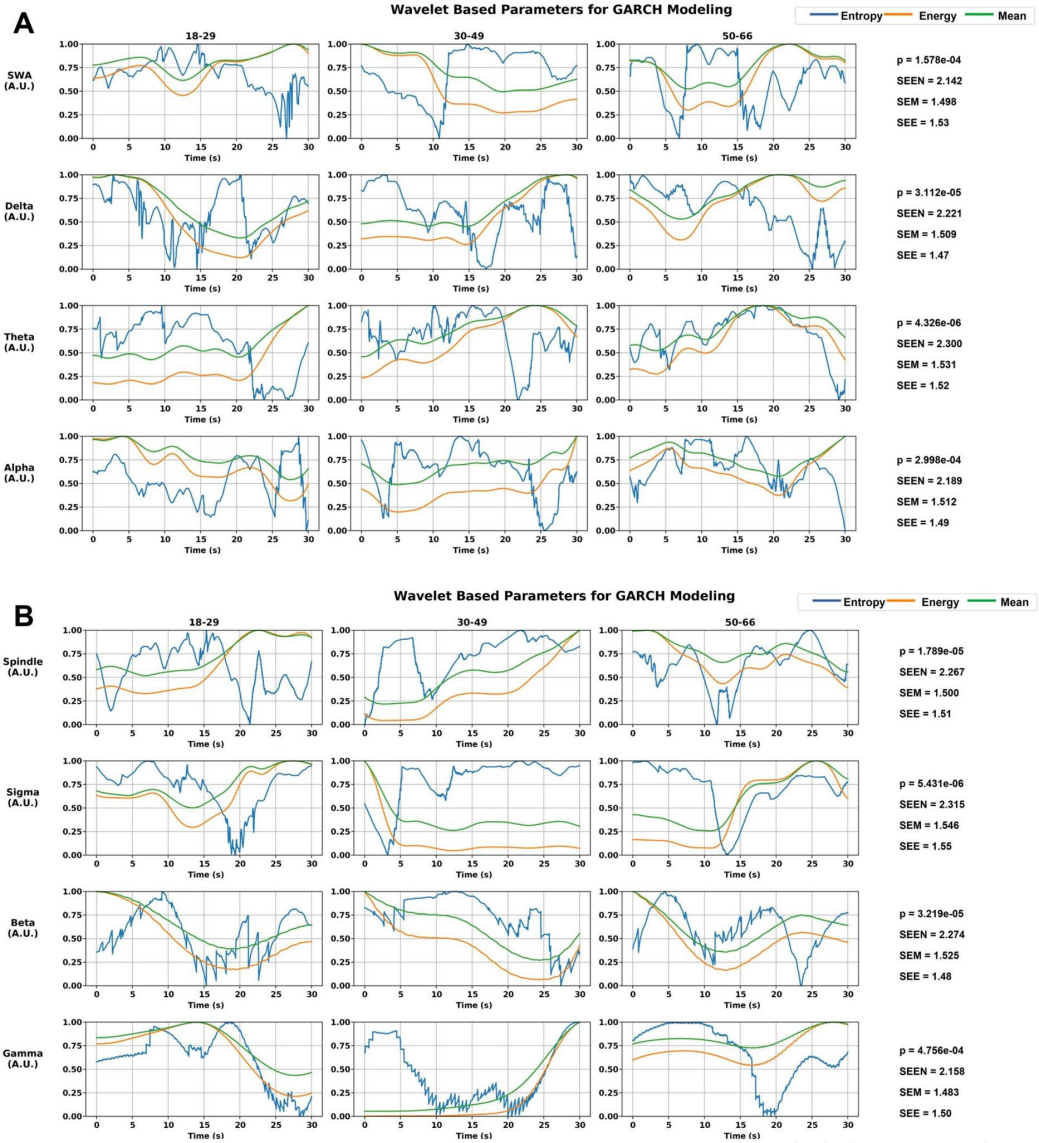
**A** Wavelet-based parameters for GARCH modeling across frequency bands and age scales. Wavelet signal mean, energy, and entropy parameterization of multimodal sleep signals represented in blue, green, and orange, respectively, across alpha, theta, delta, and SWA frequency bands and age scales. Significant *p*-values are provided for each age group and frequency band, indicating the statistical relevance of the observed variations. The Shannon entropy values for entropy (SEEN), mean (SEM), and energy (SEE) are reported, with SEEN demonstrating the highest values, indicating greater complexity. **B **Wavelet-based parameters for GARCH modeling across frequency bands and age scales. Wavelet signal mean, energy, and entropy parameterization of multimodal sleep signals represented in blue, green, and orange, respectively, across gamma, beta, sigma, and spindle frequency bands and age scales. Significant *p*-values are provided for each age group and frequency band, indicating the statistical relevance of the observed variations. The Shannon entropy values for entropy (SEEN), mean (SEM), and energy (SEE) are reported, with SEEN demonstrating the highest values, indicating greater complexity

Wavelet signal entropy was chosen as the primary input for GARCH modeling and is based on several observations. Firstly, entropy exhibited the highest degree of variability and stochasticity compared to mean and energy, reflecting the chaotic nature of neural signals during different sleep stages. This characteristic makes entropy particularly useful for capturing dynamic changes and transitions in neural states [28, 56]. Entropy is sensitive to the complexity of the neural signals and can detect subtle variations that other features might miss. By incorporating entropy into the GARCH model, we aim to gain deeper insights into the volatility and stability of neural dynamics, providing a more detailed understanding of sleep transitions and the effects of external interventions, such as pharmacological agents. Figure 4A and B present the wavelet-based parameters used for GARCH modeling of EEG signals across age groups and frequency bands. Signal entropy, mean, and energy were extracted using CWT, followed by computation of the Shannon entropy values for these parameters, represented as “SEEN (Shannon entropy of wavelet magnitude entropy),” “SEM (Shannon entropy of wavelet magnitude mean),” and “SEE (Shannon entropy of wavelet magnitude energy).”

#### Volatility measures

We employed the GARCH model to analyze the features derived from the multimodal tensor, quantifying neural signal volatility across different sleep stages, age groups, and conditions involving sleep aid usage (“GARCH modeling” section). This allowed us to determine periods of heightened volatility, indicating transition states or alterations in neural dynamics. To further examine these findings, we conducted a multiway ANOVA with Bonferroni correction, evaluating the statistical significance of the influences of age, sleep stage, frequency band, sensor location, and modality type on GARCH model-derived volatility measures.

**Fig. 5:**
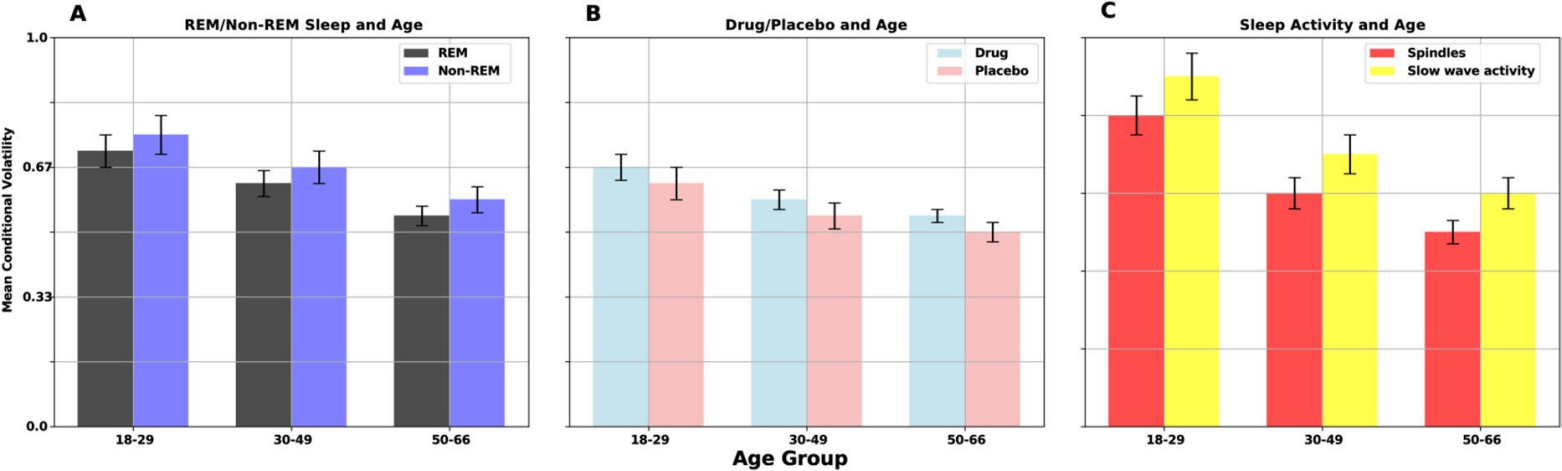
Mean conditional volatility across age groups and sleep stages for REM/non-REM sleep, drug/placebo conditions, and specific sleep activities. **A** Mean conditional volatility for REM and non-REM sleep across different age groups. **B** Mean conditional volatility for drug and placebo conditions across age groups. **C** Mean conditional volatility for spindle activity and slow wave activity across age groups. Significant differences are shown, *p* < 0.05

Additionally, we assessed mean conditional volatility across various age groups, sleep stages, and conditions (REM/non-REM, sleep aid versus placebo, and specific sleep activities), offering insights into the variability and stability of neural signals and the effects of pharmacological interventions on sleep throughout the lifespan. Figure 5 presents mean conditional volatility, demonstrating the effects of sleep aid versus placebo across REM and non-REM sleep stages and age groups, and highlights how sleep aids differently modulate neural volatility based on age and sleep stage. Supplemental Figures S1 and S2 provide an in-depth view of the variability and stability of neural signals, showcasing differences in mean conditional variance. Supplemental Figure S1 illustrates mean volatility trends across different age groups and sleep stages for both placebo and drug conditions. Supplemental Table S1 details the comparison of mean conditional volatility across various frequency bands, sleep stages, and age groups. This thorough analysis clarifies the age-related changes in neural signal variability and the influence of different sleep stages on these changes.

#### Causality analysis of volatility measures

**Fig. 6:**
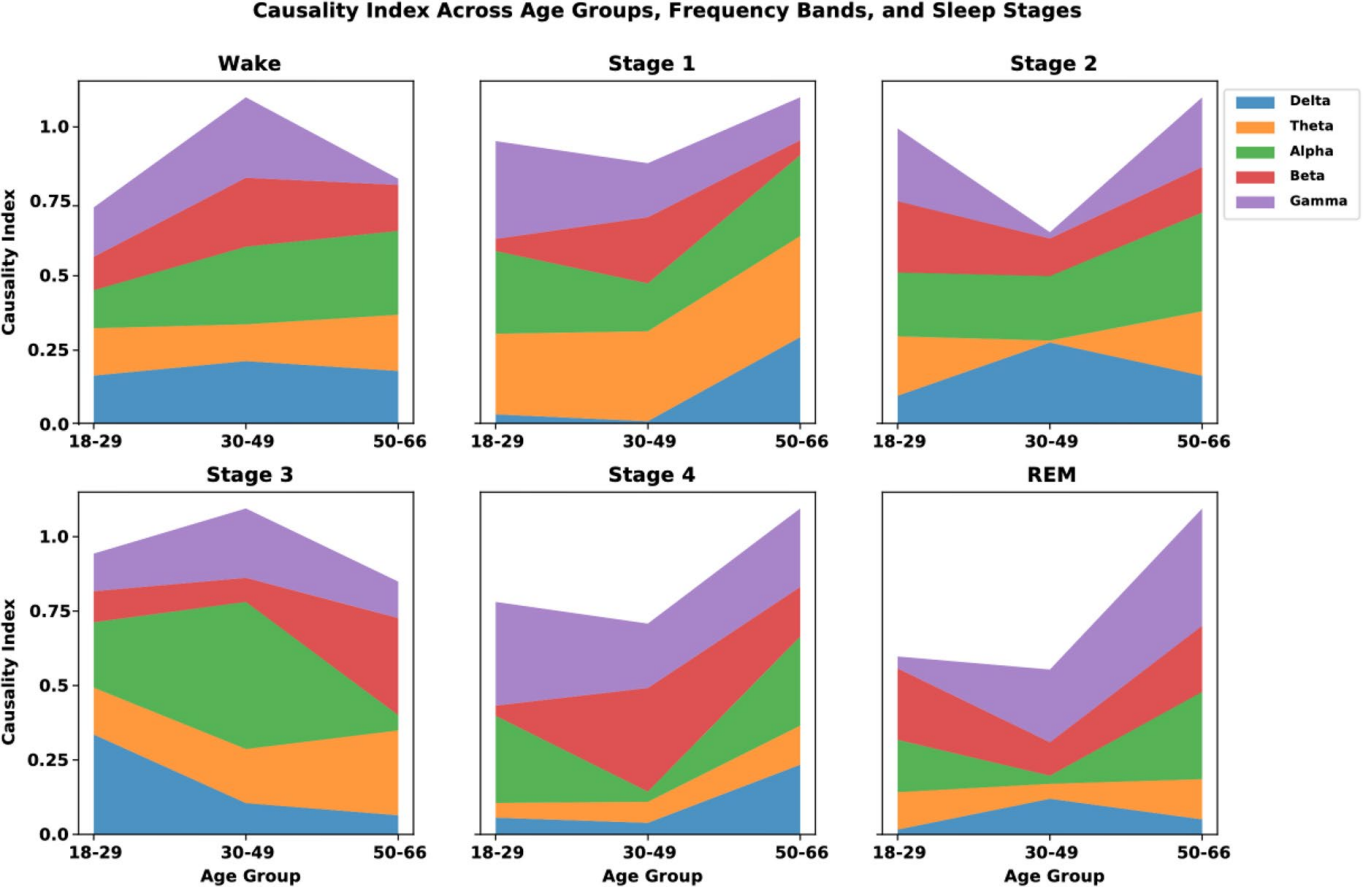
Causality index across age groups, frequency bands, and sleep stages. The causality index (CI) for age groups, frequency bands, and sleep stages is presented. In the delta band, the CI decreases with age across all sleep stages. The theta band shows consistent indices across age groups, with slight increases observed in the older age group during stage 3 and REM sleep. The alpha band exhibits moderate variability across age groups, with noticeable peaks in the 50–66 age group during wake and stage 2. The beta band reveals an increase in causality with age, particularly in stage 1 and REM sleep. The gamma band demonstrates the highest causality index in the youngest age group (18–29) during stage 2 and REM, indicating significant directional influences of neural signals in these conditions

In this section, we examine the causal relationships underlying the volatility measures using mean conditional variance and transfer entropy. Causality analysis identifies how different sleep stages, age groups, and frequency bands interact and influence neural dynamics given the effects of aging and pharmacological interventions on these processes. As shown in Fig. 6, delta and theta bands during deep sleep stages (stage 3 and REM) in the 50–66-year-old group demonstrate a higher mean causality index, indicating stronger and more stable directional signal influence. This suggests that neural activity in these frequency bands becomes more synchronous and stable with age during deep sleep stages. Conversely, the alpha and beta bands show greater variability in the causality index across different age groups and sleep stages. This variability reflects less consistent directional interactions and suggests that neural processes in these bands are more dynamic and less synchronized. Specifically, the findings indicate that neural stability and synchrony are more pronounced in the delta and theta bands during deep sleep stages in older adults, while the alpha and beta bands remain variable and dynamic. We summarize these trends and observations in Table 3 highlighting the mean power values for various frequency bands within each sleep stage and age group, offering insights into the age-related changes in sleep architecture. Table S3 in the supplemental section provides a comprehensive summary of EEG frequency band power across various sleep stages and age groups, presenting the mean power values for frequency bands within each sleep stage and age group, and Table S4 in the supplemental section summarizes key observations.

**Table 3:** Mean EEG frequency band power across sleep stages and age groups. Mean EEG frequency band power and key trends across different sleep stages and age groups. Trends include increased power in REM sleep for older age groups, decline in alpha power in stage 1 sleep for older age groups, high theta activity in younger adults during stage 2 sleep, consistent high delta and gamma activity across all age groups during stage 3 sleep, and consistently high beta and gamma activity during wakefulness across all age groups

Sleep stage	Frequency band	18–29 Age group	30–49 Age group	50–66 Group	Trend
REM	Delta	0.415	0.410	0.5	Consistent across all groups
Stage 1	Theta	0.612	0.717	0.612	High in the 30–49 age group
Stage 2	Alpha	0.577	0.405	0.439	Decline in the 18–29 to 50–66 age group
Stage 3	Gamma	0.312	0.397	0.381	Consistent across all age groups
Stage 4	Beta	0.511	0.625	0.455	Slight increase in the 30–49 age group
Wake	Beta	0.378	0.571	0.579	Consistent across all age groups
Stage 1	Spindle	0.315	0.300	0.411	Low across all groups
Stage 2	SWA	0.350	0.401	0.305	Slight increase in the 30–49 age group

#### Statistical analysis of signal volatilities

**Fig. 7:**
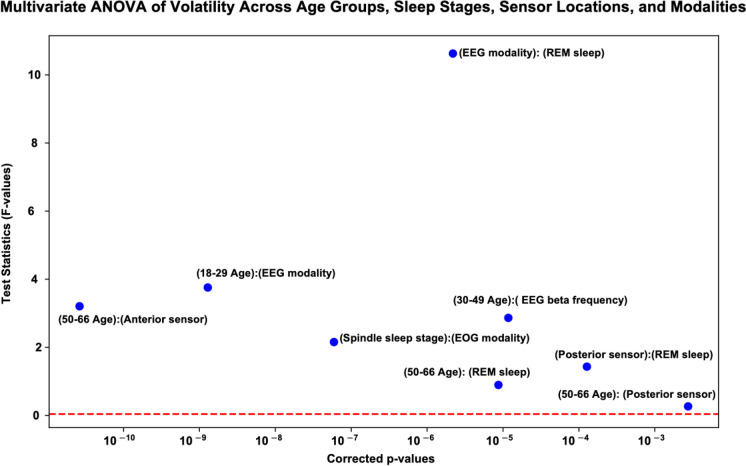
Multivariate ANOVA (MANOVA) of volatility across age groups, sleep stages, sensor locations, and modalities. Results of a multivariate ANOVA (MANOVA) analysis of signal volatility obtained from GARCH modeling highlight significant interactions between age groups, sleep stages, sensor locations, and modalities. The test statistics (*F*-values) are plotted against corrected *p*-values, with the red dashed line indicating the significance threshold for corrected *p*-values. Significant interactions include high volatility in the anterior sensors for the 50–66 age group, notable volatility in EEG signals for the 18–29 age group, and high variability in EOG signals during the spindle sleep stage. Additionally, significant volatility in the beta frequency band of EEG signals for the 30–49 age group and high variability in neural activity during REM sleep for the 50–66 age group is seen. The 50–66 age group shows significant volatility in the posterior sensors, while high volatility in posterior sensors is observed during REM sleep. The highest volatility is observed in EEG signals during REM sleep across all age groups

Multivariate ANOVA (MANOVA) after applying GARCH modeling was performed to capture the volatility of neural signals. This analysis aimed to identify significant interactions between age groups, sleep stages, and signal modalities (EEG, EMG, EOG) by examining the volatilities in neural dynamics. Figure 7 illustrates MANOVA results, showing the test statistics (*F*-values) and corrected *p*-values for various conditions. The significant interactions are highlighted with *p*-values below the threshold (indicated by the red dashed line). Notably, the EEG modality during REM sleep exhibited the highest test statistic, indicating a substantial effect on signal volatility. Other significant interactions include the anterior sensor in the 50–66 age group, the EEG modality in the 18–29 age group, and the theta frequency in the 30–49 age group. These findings highlight the interplay between age, sleep stages, sensor locations, and modalities in influencing neural signal volatility. By examining these interactions, we gain valuable insights into the variability and stability of neural signals across different conditions.

#### Mean volatility across age and sleep stages

**Fig. 8:**
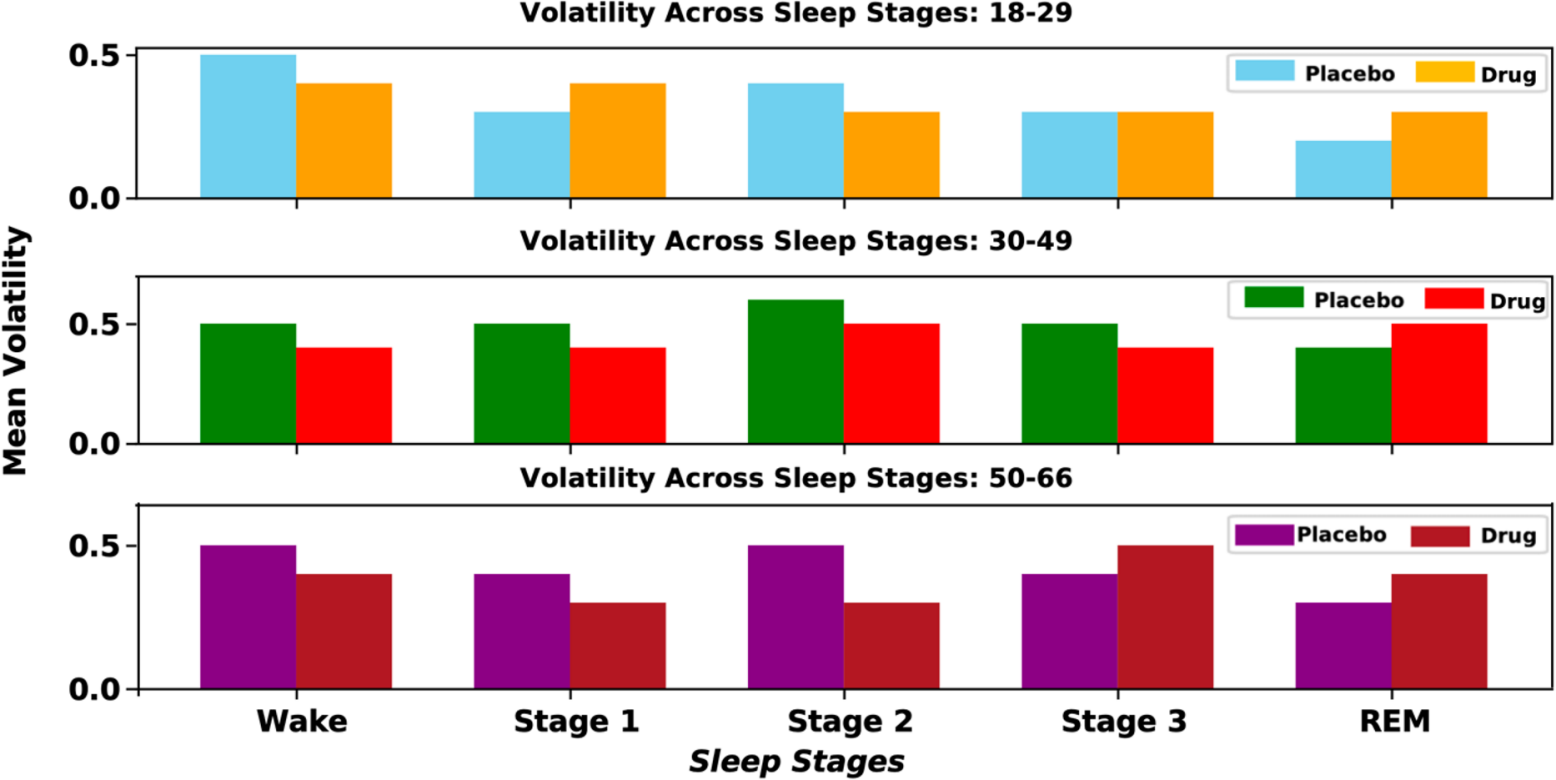
Mean volatility across sleep stages across age groups under placebo and drug conditions. Mean volatility in sleep stages (wake, stage 1, stage 2, stage 3, and REM) across age groups is shown and the comparison highlights the variability in sleep stage transitions and the impact of the drug intervention on sleep stability across the different age groups

Table 4 presents the mean volatility for both placebo and drug conditions across the 18–29, 30–49, and 50–66 age groups and various sleep stages: wake, stage 1, stage 2, stage 3, and REM sleep, indicating that temazepam generally stabilizes volatility, particularly in stage 2, across age groups, with some increased volatility in the 50–66 age group. Particularly in the wake stage, volatility fluctuates across age groups without a clear trend. In stage 1, the placebo condition shows higher volatility in the 30–49 age group, while the drug condition remains relatively stable across all ages. Stage 2 shows a decrease in volatility with age for both conditions, with a slight increase under the drug condition in the 50–66 age group. Stage 3 volatility is generally stable, with a slight increase in the 50–66 age group under the drug condition. REM sleep shows minimal differences between conditions, with slight fluctuations across age groups. Figure 8 visualizes these trends.

**Table 4:** Mean volatility for placebo and drug conditions across different sleep stages and age groups. Mean volatility values for both placebo and sleep aid across age groups, sleep stages, and the wake stage (wake (W), stage 1, stage 2, stage 3, and REM (R)). In the wake stage (W), the volatility values fluctuate significantly across age groups for both placebo and drug conditions. Mean volatility observations suggest that temazepam generally stabilizes volatility across sleep stages and age groups, particularly in deeper sleep stages such as stage 2 and stage 3. This stabilization effect is more pronounced in older age groups, indicating the potential efficacy of temazepam in mitigating age-related increases in sleep stage volatility

Age group	W (placebo, drug)	Stage 1 (placebo, drug)	Stage 2 (placebo, drug)	Stage 3 (placebo, drug)	R (placebo, drug)
18–29	(0.5, 0.4)	(0.3, 0.4)	(0.4, 0.3)	(0.3, 0.3)	(0.2, 0.3)
30–49	(0.5, 0.4)	(0.5, 0.4)	(0.6, 0.5)	(0.5, 0.4)	(0.4, 0.5)
50–66	(0.5, 0.4)	(0.4, 0.3)	(0.5, 0.3)	(0.4, 0.5)	(0.3, 0.4)

## Discussion

Modeling sleep multimodal multiscale neural dynamics provides a methodological framework to examine signal volatility across the lifespan, thereby parameterizing the assessment of pharmacological interventions and mitigating age-related effects in sleep quality. Here, we focus on EEG frequency bands and specific sleep-related activities to hypothesize that sleep aids (i.e., temazepam) mitigate age-related declines in deep and restorative sleep, evidenced by changes in delta waves, sleep spindles, and slow wave activity (SWA). Our analysis involved tensorizing multimodal EEG-EMG-EOG signals followed by continuous wavelet transform (CWT) feature extraction representing delta (0.5–4 Hz), theta (4–8 Hz), alpha (8–12 Hz), beta (12–30 Hz), gamma (30–50 Hz) frequency bands, sleep spindles (11–16 Hz), and SWA (0.5–2 Hz). A Generalized Autoregressive Conditional Heteroskedasticity (GARCH) model was implemented to quantify feature volatility, followed by statistical testing to identify significant interactions between age groups, sleep stages, and signal modalities (Fig. 1). GARCH modeling is well-suited to determine time-varying volatility associated with sleep stage transitions, as it accounts for dynamic variance (i.e., heteroskedasticity) present in neural signal dynamics [31, 33]. Contextually, GARCH effectively identifies periods of instability that correspond to transitions between sleep stages. Specifically, in our analysis, delta waves showed a significant age-related decline, noticeable in the 30–49 and 50–66 age groups (Figs. 2 and 3). Our results highlight significant age-related declines in delta power, spindle activity, and SWA in the placebo group, indicating reduced deep and restorative sleep typically observed with aging. When examining temazepam’s effects, stable delta power across age groups was noticed, suggesting that sleep aids may help preserve sleep in older adults. Sleep spindles, associated with sleep stability and memory consolidation, also showed a significant decline in the placebo group with age $(p = 6.737e-02)$. However, the sleep aid group exhibited a significant increase in spindle activity with age $(p = 1.025e-04)$, indicating that sleep aids enhance spindle activity, potentially improving sleep stability and memory processes in older adults. SWA, indicative of deep restorative sleep, showed a significant decline in the placebo group with age $(p = 2.146e-02)$. In the sleep aid group, SWA was preserved across age groups $(p = 7.293e-03)$, suggesting that sleep aids may help maintain deep sleep in older age groups.

The administration of sleep aid (i.e., temazepam) demonstrated a stabilizing effect on neural signal volatility across different age groups and sleep stages. The sleep aid group showed preserved delta power, enhanced spindle activity, and maintained SWA across age groups, suggesting the potential of sleep aids to mitigate age-related declines in sleep quality and stability. The stability effect of temazepam was particularly pronounced in the 50–66 age group during stage 2 sleep, where the mean volatility values were significantly lower compared to the placebo condition, suggesting temazepam enhances the stability of sleep architecture in older adults, potentially leading to more restorative sleep and better overall sleep quality. By applying GARCH modeling, we quantify the dynamic volatility of neural signals across sleep stages and age groups. Higher fluctuations of mean conditional volatility (MCV) in the 50–66 age group compared to the 18–29 and 30–49 age groups were observed, particularly during transitions from stage 3 to REM sleep suggesting aging may be linked to less stable neural dynamics and more frequent sleep stage transitions. Temazepam stabilizes these transitions, reducing mean conditional volatility and potentially leading to more continuous and restorative sleep. Figure 4 presents the wavelet-based parameters for GARCH modeling, displaying trends in wavelet signal entropy, energy, and mean across different frequency bands and age groups. Each subplot represents a specific frequency band and age group with entropy (blue line), energy (orange line), and mean (green line) plotted over time. Significant *p*-values and Shannon entropy values (“SEEN,” “SEM,” and “SEE” for wavelet signal entropy, wavelet signal mean, and wavelet signal energy, respectively) are provided for each subplot. The results show that in the delta band for the 18–29 age group, entropy (SEEN) is 2.221, while energy (SEE) and mean (SEM) are 1.47 and 1.509, respectively. Similar patterns are observed across other frequency bands and age groups, suggesting that entropy is the most sensitive measure for capturing the intricate variations in neural signals. In the theta band, entropy values are particularly high for the 30–49 age group and the 50–66 age group, indicating increased complexity in these age ranges. The alpha band shows a significant peak in entropy for the 30–49 age group (SEEN = 2.189), highlighting distinct neural dynamics in this cohort. Spindle activity exhibits higher entropy values in the 18–29 and 30–49 age groups, reflecting the nuanced neural processes associated with sleep spindles in younger adults. Sigma and beta bands demonstrate increased entropy in the 30–49 and 50–66 age groups, while gamma-band entropy peaks in the 50–66 age group, emphasizing the variability and complexity in high-frequency neural oscillations with aging. These findings underscore the utility of wavelet-based entropy measures in capturing the dynamic complexity of neural signals across different sleep stages and age groups. The higher entropy values indicate more complex and less predictable signal characteristics, making entropy a valuable feature for GARCH modeling and the analysis of neural volatility during sleep transitions.

Figure 5 shows the mean conditional volatility across different age groups and sleep stages, demonstrating that REM sleep has higher volatility compared to non-REM sleep, with significant differences indicating more stable neural dynamics in non-REM sleep. Temazepam exhibits lower volatility compared to the placebo condition, particularly in the 50–66 age group, suggesting the stabilizing effects of sleep aids. Figure 6 illustrates the causality index (CI) across age groups, frequency bands, and sleep stages. Delta and theta bands during deep sleep stages in the 50–66 age group show higher mean causality indices, indicating stronger and more stable directional signal influence. Conversely, alpha and beta bands show greater variability, reflecting less consistent directional interactions and suggesting that neural processes in these bands are more dynamic and less synchronized. The gamma band exhibits the highest CI across all age groups, as opposed to lower frequency envelopes (i.e., delta and theta) suggesting slower oscillations play a less prominent role in influencing neural activity. In sleep stages, there is an increase in the CI of the gamma band across all age groups, with a marked rise in the 50–66 age group. Likewise, alpha and beta bands maintain strong CI values, indicating the continued importance of these frequencies in early sleep stages. However, slower oscillations (i.e., delta band) have a decrease in CI, particularly in older groups, suggesting a reduced influence of slow-wave activity at this stage. Figure 7 presents the results of multivariate ANOVA analysis examining the volatility of neural signals across various age groups, sleep stages, sensor locations, and modalities. The *x*-axis displays corrected *p*-values on a logarithmic scale, with lower values indicating higher statistical significance after multiple testing corrections, while the *y*-axis shows the test statistics (*F*-values), where higher values denote stronger effects or greater differences between groups. The red dashed line represents the threshold for statistical significance, and points above this line are considered statistically significant after Bonferroni multiple comparisons corrections. Significant findings include notable differences in volatility in the EEG modality during REM sleep for the 18–29 and 30–49 age groups and in the EEG beta frequency for the 30–49 age group. Additionally, significant effects were observed in the spindle sleep stage within the EOG modality and at anterior sensor locations for the 50–66 age group. The posterior sensor locations also showed significant volatility differences during REM sleep for the 50–66 age group. Table 4 and Fig. 8 compare the mean volatility values for placebo and drug conditions across different age groups and sleep stages. The wake stage shows significant fluctuations in mean volatility across age groups for both conditions, while sleep stages 1 and 2 demonstrate stability, suggesting that temazepam generally stabilizes volatility across sleep stages and age groups, particularly in deeper sleep stages and older adults. Utilizing the tensor-based framework to integrate multi-dimensional data using wavelet-based feature extraction and statistical modeling, i.e., Generalized Autoregressive Conditional Heteroskedasticity (GARCH), enhances the resolution and sensitivity of detecting subtle neural transition sleep patterns. Future work exploring the long-term effects of sleep aids on neural dynamics and sleep architecture across age groups in a longitudinal study, including a broader range of sleep aids and dosages, can determine the most effective interventions for aging sleep disorders.

## Conclusion

This study provides a comprehensive framework for analyzing multimodal sleep signals and addresses age-related changes in sleep architecture. It aims to deepen our understanding of the neural dynamics underlying sleep transitions, focusing on the effects of aging and sleep aids (i.e., temazepam). By integrating multimodal EEG, EMG, and EOG data, the study employs a comprehensive methodology combining tensor multimodal representation, wavelet-based feature extraction, and Generalized Autoregressive Conditional Heteroskedasticity (GARCH) volatility modeling. Our findings highlight significant age-related declines in neural signal stability, particularly in delta, spindle, and slow wave activity frequency bands, with the most pronounced decreases observed in the 30–49 and 50–66 age groups, indicating reduced deep and restorative sleep. Temazepam administration demonstrated a stabilizing effect on neural signal volatility, preserving delta power, enhancing spindle activity, and maintaining slow wave activity across the lifespan, suggesting sleep aids can mitigate age-related declines in sleep quality. GARCH modeling provided quantification of neural signal volatility, revealing higher fluctuations in older age groups during transitions from deep sleep to REM and a causality index of 0.80 from frontal to occipital regions during REM sleep state transitions. Multivariate ANOVA (MANOVA) analysis identified significant interactions between age groups, sleep stages, and signal modalities. These results suggest that sleep aids stabilize neural dynamics and improve sleep quality as age increases and provide the framework for future work to examine long-term effects of sleep aids and their potential health benefits longitudinally across the lifespan.

## Supplementary Information

Below is the link to the electronic supplementary material.

Supplementary file1 (DOCX 461 KB)

## Data Availability

The data and code to accompany this work can be obtained upon reasonable request.
