# Supplementary material for: Multiscale neural dynamics in sleep transition volatility across age scales: a multimodal EEG-EMG-EOG analysis of temazepam effects
Source: GeroScience. 2024 Sep 14;47(1):205–26. doi: 10.1007/s11357-024-01342-6 (PMC11872868; doi:10.1007/s11357-024-01342-6)
Supplement: Supplementary file 1 — Supplementary file1 (DOCX 461 KB) [file 11357_2024_1342_MOESM1_ESM.docx]

**Supplemental**

We include a detailed analysis of mean conditional variance across various sleep stages, frequency bands, and age groups to complement our primary focus on transfer entropy. Supplemental Figure S1 below presents a pairwise scatter plot matrix illustrating the mean conditional variance across different sleep stages, including slow wave activity (SWA), spindle activity, alpha waves, and beta waves. The diagonal histograms display the distribution of each variable, while the scatter plots depict the relationships between each pair of variables.


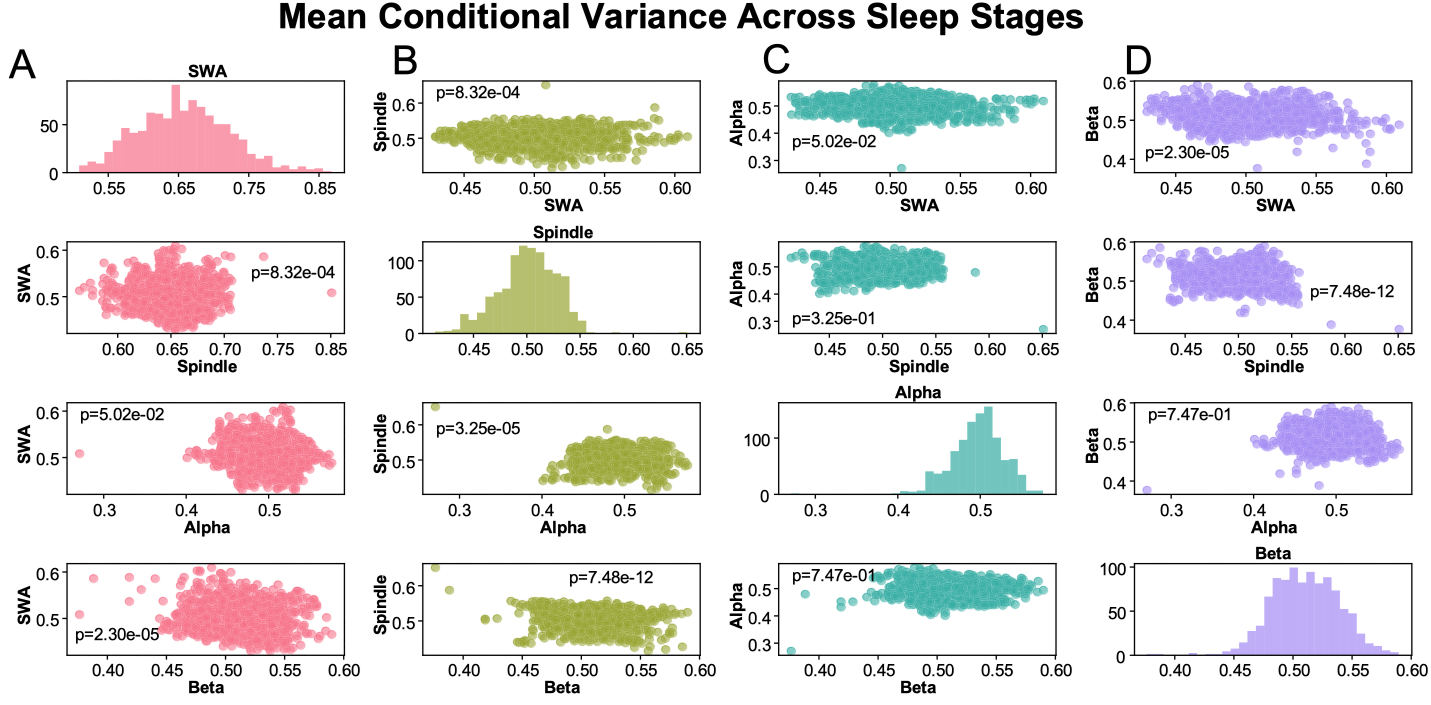


**Figure S1:** **Mean Conditional Variance Across Sleep Stages**. The distribution and scatter plots of mean conditional variance across various combinations of frequency bands and sleep stages is shown. Panel A shows the relationship between Slow Wave Activity (SWA) and spindle, alpha, and beta bands, highlighting significant differences with p-values such as $p = 8.32e-04$ for SWA and spindle, and $p = 2.30e-05$ for SWA and Beta. Panel B illustrates spindle activity and its relationship with SWA, alpha, and beta bands, with notable p-values like $p = 8.32e-04$ for spindle and SWA, and $p = 7.48e-12$ for spindle and beta. Panel C focuses on alpha activity, showing its variance distribution and relationships with SWA, spindle, and beta bands, with significant p-values such as $p = 5.02e-02$ for alpha and SWA. Panel D presents Beta activity and its correlation with SWA, spindle, and alpha bands, highlighting significant differences with p-values like $p = 2.30e-05$ for beta and SWA, and $p = 7.48e-12$ for beta and spindle activity. These plots demonstrate the variability in neural signal stability across different sleep stages and frequency bands, providing valuable insights into the predictability and risk associated with neural dynamics.

Figure S1 presents a comprehensive analysis of the relationships between different EEG frequency bands (slow wave activity (SWA), spindle, alpha, beta) across various sleep stages. Panel A shows the histogram of SWA and its scatter plots with spindle, alpha, and beta bands. The histogram illustrates the distribution of SWA values, predominantly ranging between 0.55 and 0.85. The scatter plots indicate significant correlations between SWA and other frequency bands, particularly with spindle $(p=8.32e-04)$ and beta $(p=2.30e-05)$ bands, suggesting strong interactions between these neural activities during sleep. Panel B focuses on the Spindle band, presenting its histogram and scatter plots with SWA, alpha, and beta bands. The spindle band shows significant correlations with SWA $(p=8.32e-04)$ and beta $(p=7.48e-12)$ bands. These results indicate that spindle activity is closely related to both slow wave and high-frequency beta activity, which could be critical for understanding sleep stability and memory consolidation. Panel C presents the alpha band, with a histogram and scatter plots with SWA, spindle, and beta bands. Alpha activity shows a moderate correlation with SWA $(p=5.02e-02)$ but less pronounced interactions with spindle $(p=3.25e-01)$ and beta $(p=7.47e-01)$ bands. This suggests that alpha activity, typically associated with relaxation and light sleep, has a distinct dynamic compared to the other frequency bands. Panel D illustrates the beta band, with its histogram and scatter plots showing correlations with SWA, spindle, and alpha bands. The beta band displays significant correlations with SWA $(p=2.30e-05)$ and spindle $(p=7.48e-12)$ bands, reinforcing the observation that beta activity is intricately linked with both slow wave and spindle activities, potentially reflecting the neural processes underlying cognitive and sensory functions during sleep. Overall, these findings provide a detailed view of how different EEG frequency bands interact during various sleep stages, highlighting significant correlations and the complexity of neural dynamics during sleep. The specific p-values indicate the statistical significance of these interactions, emphasizing the robust relationships between these frequency bands across different sleep stages.

Table S1 below provides a detailed comparison of mean conditional volatility across various frequency bands, sleep stages, and age groups. This comprehensive analysis helps to elucidate the age-related changes in neural signal variability and the effects of different sleep stages on these changes. Supplemental Table S2 presents the overall trends observed across the different frequency bands and signals for various age groups, providing a clearer understanding of how these values change with age and sleep states. Supplemental Table S3 provides a comprehensive summary of EEG frequency band power across various sleep stages and age groups. The table presents the mean power values for different frequency bands (alpha, beta, delta, gamma, theta) within each sleep stage (REM, Stage 1, Stage 2, Stage 3, Wake) and across age groups. Each row represents a specific frequency band within a given sleep stage, while the columns display the corresponding power values for each age group, along with the overall population mean. This detailed breakdown helps to understand the age-related changes in sleep architecture and EEG frequency band power. Supplemental Table S4 summarizes key observations.

| **Table S1: Mean conditional volatility (MCV) for frequency bands and electrophysiological signals (SWA, Delta, Theta, Gamma, Alpha, Beta, Spindle, Sigma, and EOG) across different age groups.** Trends and variations in MCV across age groups. Note that MCV declines with increasing age across all frequency bands and sleep stages. Notably, SWA and Spindle activity are absent during wakefulness, and Delta and Theta waves, while present during wakefulness, exhibit higher values during sleep, reflecting their role in sleep-related neural processes.   \| **Frequency Bands and Signals** \| **18-29 Wake** \| **18-29 NREM** \| **18-29 REM** \| **30-49 Wake** \| **30-49 NREM** \| **30-49 REM** \| **50-66 Wake** \| **50-66 NREM** \| **50-66 REM** \| \| --- \| --- \| --- \| --- \| --- \| --- \| --- \| --- \| --- \| --- \| \| **SWA** \| N/A \| 0.76 \| 0.78 \| 0.81 \| 0.75 \| 0.77 \| 0.74 \| 0.7 \| 0.71 \| \| **Delta** \| 0.74 \| 0.71 \| 0.72 \| 0.68 \| 0.66 \| 0.67 \| 0.65 \| 0.62 \| 0.63 \| \| **Theta** \| 0.63 \| 0.64 \| 0.65 \| 0.59 \| 0.57 \| 0.58 \| 0.54 \| 0.52 \| 0.53 \| \| **Gamma** \| 0.47 \| 0.44 \| 0.45 \| 0.43 \| 0.42 \| 0.42 \| 0.39 \| 0.38 \| 0.38 \| \| **Alpha** \| 0.33 \| 0.35 \| 0.36 \| 0.31 \| 0.32 \| 0.33 \| 0.29 \| 0.3 \| 0.31 \| \| **Beta** \| 0.29 \| 0.29 \| 0.3 \| 0.28 \| 0.27 \| 0.27 \| 0.26 \| 0.25 \| 0.25 \| \| **Spindle** \| N/A \| 0.27 \| 0.28 \| 0.25 \| 0.24 \| 0.25 \| 0.23 \| 0.22 \| 0.23 \| \| **Sigma** \| 0.24 \| 0.25 \| 0.26 \| 0.23 \| 0.22 \| 0.23 \| 0.21 \| 0.2 \| 0.21 \| \| **EOG Signals** \| 0.18 \| 0.17 \| 0.19 \| 0.17 \| 0.16 \| 0.17 \| 0.15 \| 0.14 \| 0.15 \| |
| --- | --- | --- | --- | --- | --- | --- | --- | --- | --- | --- | --- | --- | --- | --- | --- | --- | --- | --- | --- | --- | --- | --- | --- | --- | --- | --- | --- | --- | --- | --- | --- | --- | --- | --- | --- | --- | --- | --- | --- | --- | --- | --- | --- | --- | --- | --- | --- | --- | --- | --- | --- | --- | --- | --- | --- | --- | --- | --- | --- | --- | --- | --- | --- | --- | --- | --- | --- | --- | --- | --- | --- | --- | --- | --- | --- | --- | --- | --- | --- | --- | --- | --- | --- | --- | --- | --- | --- | --- | --- | --- | --- | --- | --- | --- | --- | --- | --- | --- | --- | --- |

**Table S2:** **Mean Conditional Volatility (MCV) Across Frequency Bands and Age Groups Across Sleep.** Summary of the key trends and observations of mean conditional volatility (MCV) for frequency bands (SWA, Delta, Theta, Gamma, Alpha, Beta, Spindle, Sigma, EOG Signal) across age groups and sleep states. MCV values generally decrease with age, reflecting a decline in neural signal stability in older age groups. The most significant declines are observed in the SWA and Delta bands, which are essential for deep sleep and restorative processes. EOG signals exhibit the lowest MCV values overall, indicating less volatility compared to other frequency bands.

| Frequency Bands and Signals | Key Trends and Observations |
| --- | --- |
| NREM | SWA is not present during wakefulness. For NREM, MCV starts at 0.76 for the 18-29 age group, decreases slightly to 0.75 for the 30-49 age group, and further declines to 0.70 by age 50-66. In REM, MCV starts at 0.78 for the 18-29 age group, decreases to 0.77 for the 30-49 age group, and drops further to 0.71 by age 50-66. |
| Delta | Delta MCV is 0.74 during wakefulness, 0.71 in NREM, and 0.72 in REM for the 18-29 age group. These values decrease consistently with age, reaching 0.65 during wakefulness, 0.62 in NREM, and 0.63 in REM by age 50-66. |
| Theta | Theta MCV starts at 0.63 during wakefulness, 0.64 in NREM, and 0.65 in REM for the 18-29 age group. There is a steady decline with age, with values reaching 0.54 during wakefulness, 0.52 in NREM, and 0.53 in REM by age 50-66. |
| Gamma | Gamma MCV is moderate at 0.47 during wakefulness, 0.44 in NREM, and 0.45 in REM for the 18-29 age group. It decreases gradually with age, reaching 0.39 during wakefulness, 0.38 in NREM, and 0.38 in REM by age 50-66. |
| Alpha | Alpha MCV starts at 0.33 during wakefulness, 0.35 in NREM, and 0.36 in REM for the 18-29 age group. It shows a consistent decrease with age, reaching 0.29 during wakefulness, 0.30 in NREM, and 0.31 in REM by age 50-66. |
| Beta | \|  \| \| --- \|  \| Beta MCV is 0.29 during wakefulness, 0.29 in NREM, and 0.30 in REM for the 18-29 age group. These values decrease slightly with age, reaching 0.26 during wakefulness, 0.25 in NREM, and 0.25 in REM by age 50-66. \| \| --- \| |
| Spindle | Spindle MCV is not present during wakefulness. It starts at 0.27 in NREM and 0.28 in REM for the 18-29 age group. The values decrease gradually with age, reaching 0.22 in NREM and 0.23 in REM by age 50-66. |
| Sigma | Sigma MCV is 0.24 during wakefulness, 0.25 in NREM, and 0.26 in REM for the 18-29 age group. It decreases progressively with age, reaching 0.21 during wakefulness, 0.20 in NREM, and 0.21 in REM by age 50-66. |
| EOG Signal | EOG signal MCV starts at 0.18 during wakefulness, 0.17 in NREM, and 0.19 in REM for the 18-29 age group. These values decrease with age, reaching 0.15 during wakefulness, 0.14 in NREM, and 0.15 in REM by age 50-66. |

**Table S3: EEG Frequency Band Power Across Different Sleep Stages and Age Group**. Mean EEG frequency band power across various sleep stages and age groups. Each row represents a specific frequency band (Alpha, Beta, Delta, Gamma, Theta) within a given sleep stage, and the columns display the corresponding power values for each age group, along with the overall population mean.

| **Sleep Stage** | **EEG Band** | **Age: 18-29** | **Age: 30-49** | **Age: 50-66** | **Population Mean** |
| --- | --- | --- | --- | --- | --- |
| **REM** | Alpha Band | 0.1355 | 0.2903 | 0.5500 | 0.3253 |
|  | Beta Band | 0.6001 | 0.4754 | 0.7505 | 0.6087 |
|  | Delta Band | 0.7003 | 0.5256 | 0.8306 | 0.6855 |
|  | Gamma Band | 0.8102 | 0.6807 | 0.8508 | 0.7806 |
|  | Theta Band | 0.0202 | 0.3504 | 0.5006 | 0.2904 |
| **Stage 1** | Alpha Band | 0.6906 | 0.1802 | 0.6003 | 0.4903 |
|  | Beta Band | 0.3104 | 0.3807 | 0.6005 | 0.4305 |
|  | Delta Band | 0.2906 | 0.6108 | 0.4504 | 0.4506 |
|  | Gamma Band | 0.2505 | 0.3007 | 0.5006 | 0.3506 |
|  | Theta Band | 0.6201 | 0.5604 | 0.7407 | 0.6404 |
| **Stage 2** | Alpha Band | 0.7504 | 0.5208 | 0.5806 | 0.6173 |
|  | Beta Band | 0.2905 | 0.4007 | 0.5406 | 0.4106 |
|  | Delta Band | 0.1704 | 0.2808 | 0.4705 | 0.3072 |
|  | Gamma Band | 0.5903 | 0.3907 | 0.6708 | 0.5506 |
|  | Theta Band | 0.7106 | 0.5807 | 0.7508 | 0.6807 |
| **Stage 3** | Alpha Band | 0.7306 | 0.6207 | 0.6405 | 0.6639 |
|  | Beta Band | 0.2504 | 0.6008 | 0.7407 | 0.5306 |
|  | Delta Band | 0.3305 | 0.4507 | 0.6708 | 0.4839 |
|  | Gamma Band | 0.7906 | 0.6707 | 0.8506 | 0.7706 |
|  | Theta Band | 0.5703 | 0.4106 | 0.6907 | 0.5572 |
| **Wake** | Alpha Band | 0.5605 | 0.6207 | 0.7408 | 0.6406 |
|  | Beta Band | 0.7206 | 0.7507 | 0.8108 | 0.7607 |
|  | Delta Band | 0.5804 | 0.6408 | 0.7109 | 0.6439 |
|  | Gamma Band | 0.1502 | 0.4506 | 0.5805 | 0.3938 |
|  | Theta Band | 0.4706 | 0.6507 | 0.7508 | 0.6237 |

**Table S4:** **Key Trends and Observations Across Sleep Stages and Age Groups.** Trends and observations for EEG frequency band power across different sleep stages (REM, Stage 1, Stage 2, Stage 3, Wake) and age groups (18-29, 30-49, 50-66). Notably, the REM stage shows increased power in multiple bands in the 50-66 age group, while Stage 1 shows a decline in alpha band power in older adults. Stage 2 is characterized by high theta activity in younger adults, and Stage 3 demonstrates high delta and gamma activity across all age groups. During wakefulness, beta and gamma activity remain consistently high across all ages.

| Sleep Stage | Key Trends and Observations |
| --- | --- |
| REM | Increased power across multiple bands, particularly in the 50-66 age group, with notable increases in gamma and delta band activity. |
| Stage 1 | Decline in alpha band power in the 50-66 age group. Increased beta activity in the 30-49 age group. |
| Stage 2 | Decline in alpha band power in the 50-66 age group, with high theta activity in the 18-29 and 30-49 age groups. Moderate increases in delta band power in the 50-66 age group. |
| Stage 3 | High delta and gamma activity across all age groups, with a slight decline in theta band power in the 30-49 age group. |
| Wake | \| Peak in theta band power in the 30-49 age group. Consistently high beta and gamma activity across all age groups. Alpha band power increases slightly in the 50-66 age group. \| \| --- \|  \|  \| \| --- \| |
